# Supplementary material for: Post-acute sequelae of SARS-CoV-2 with clinical condition definitions and comparison in a matched cohort
Source: Nat Commun. 2022 Oct 12;13:5822. doi: 10.1038/s41467-022-33573-6 (PMC9556630; doi:10.1038/s41467-022-33573-6)
Supplement: Supplementary file 1 — Supplementary Information [file 41467_2022_33573_MOESM1_ESM.pdf]

Extended Data

Supplementary Table 1: Risk and Cumulative Incidence of All CCS Categories

CCS Category and Time Period	Case Cumulative Incidence	Control Cumulative Incidence	Risk Ratio [95%CI]
Total - Any condition			
Late	26.1%	25.2%	1.04[1.01,1.07]*
Acute and Persistent	20.4%	22.1%	0.92[0.89,0.95]*
Pre-existing Conditions	46.1%	44.2%	1.04[1.02,1.06]*
Total - PASC Related Conditions			
Late	13.6%	12.1%	1.12[1.08,1.16]*
Acute and Persistent	4.1%	2.5%	1.60[1.48,1.72]*
Pre-existing Conditions	30.7%	29.0%	1.06[1.03,1.08]*
Abdominal pain [a]			
Late	1.8%	1.7%	1.05[0.95,1.17]
Acute and Persistent	0.2%	0.2%	0.87[0.63,1.20]
Pre-existing Conditions	3.4%	3.4%	1.00[0.93,1.07]
Adjustment disorders			
Late	1.0%	1.0%	1.01[0.88,1.15]
Acute and Persistent	0.2%	0.1%	1.28[0.91,1.79]
Pre-existing Conditions	0.8%	1.0%	0.83[0.71,0.96]*
Adverse effects of medical care			
Late	0.0%	0.0%	0.75[0.21,2.73]
Acute and Persistent		0.0%	
Pre-existing Conditions		0.0%	
Anosmia [a]			
Late	0.3%	0.1%	3.88[2.79,5.40]*
Acute and Persistent	0.0%	0.0%	0.50[0.11,2.28]
Pre-existing Conditions	0.0%	0.0%	1.25[0.31,5.00]
Anxiety disorders [a]			
Late	1.1%	1.1%	1.01[0.89,1.15]
Acute and Persistent	0.2%	0.3%	0.83[0.63,1.10]
Pre-existing Conditions	2.8%	3.6%	0.78[0.72,0.84]*
Birth trauma			
Pre-existing Conditions	0.0%		
Cancer			
Late	0.4%	0.4%	0.87[0.70,1.09]
Acute and Persistent	0.0%	0.3%	0.10[0.05,0.19]*
Pre-existing Conditions	1.7%	2.2%	0.75[0.67,0.83]*
Cancer of bladder			
Late		0.0%	
Acute and Persistent		0.0%	

Pre-existing Conditions	0.0%	0.1%	0.49[0.24,1.00]*
Cancer of esophagus			
Late		0.0%	
Acute and Persistent		0.0%	
Pre-existing Conditions	0.0%	0.0%	0.56[0.12,2.57]
Cancer of head and neck			
Late	0.0%	0.0%	0.68[0.19,2.44]
Acute and Persistent		0.0%	
Pre-existing Conditions	0.0%	0.1%	0.52[0.26,1.03]
Cancer of other female genital organs			
Late		0.0%	
Acute and Persistent		0.0%	
Pre-existing Conditions	0.0%	0.0%	1.36[0.50,3.69]
Cancer of other male genital organs			
Late		0.0%	
Acute and Persistent		0.0%	
Cancer of other urinary organs			
Pre-existing Conditions		0.0%	
Cancer of pancreas			
Late	0.0%	0.0%	1.00[0.19,5.15]
Acute and Persistent		0.0%	
Pre-existing Conditions	0.0%	0.0%	0.58[0.16,2.02]
Cancer of testis			
Acute and Persistent		0.0%	
Pre-existing Conditions	0.0%	0.0%	1.43[0.42,4.88]
Cancer of thyroid			
Late	0.0%	0.0%	0.23[0.03,1.76]
Acute and Persistent		0.0%	
Pre-existing Conditions	0.1%	0.1%	0.54[0.33,0.90]*
Cancer of uterus			
Late	0.0%	0.0%	1.11[0.34,3.61]
Acute and Persistent		0.0%	
Pre-existing Conditions	0.1%	0.1%	0.98[0.60,1.61]
Cancer; other respiratory and intrathoracic			
Late		0.0%	
Pre-existing Conditions	0.0%	0.0%	2.50[0.16,39.97]
Cardiac and circulatory congenital anomalies			
Late	0.0%	0.0%	1.02[0.47,2.22]
Acute and Persistent	0.0%	0.0%	0.50[0.06,4.28]
Pre-existing Conditions	0.1%	0.1%	0.70[0.41,1.19]

Cardiac dysrhythmias [a]			
Late	0.9%	0.7%	1.25[1.08,1.45]*
Acute and Persistent	0.3%	0.2%	1.90[1.45,2.49]*
Pre-existing Conditions	1.6%	1.5%	1.02[0.91,1.14]
Chronic ulcer of skin			
Late	0.1%	0.1%	0.93[0.55,1.55]
Acute and Persistent	0.0%	0.0%	1.15[0.58,2.29]
Pre-existing Conditions	0.2%	0.2%	1.23[0.91,1.66]
Coagulation and hemorrhagic disorders			
Late	0.3%	0.3%	1.02[0.78,1.32]
Acute and Persistent	0.0%	0.0%	1.25[0.64,2.43]
Pre-existing Conditions	0.2%	0.3%	0.88[0.66,1.16]
Coma; stupor; and brain damage			
Late	0.0%	0.0%	1.00[0.51,1.95]
Acute and Persistent	0.0%	0.0%	2.50[0.35,17.75]
Pre-existing Conditions	0.0%	0.0%	1.87[0.65,5.40]
Conditions associated with dizziness or vertigo [a]			
Late	1.7%	1.6%	1.05[0.94,1.16]
Acute and Persistent	0.2%	0.2%	1.08[0.82,1.44]
Pre-existing Conditions	3.6%	3.4%	1.04[0.96,1.12]
Cystic fibrosis			
Pre-existing Conditions	0.0%		
Deficiency and other anemia			
Late	0.7%	0.6%	1.20[1.01,1.41]*
Acute and Persistent	0.3%	0.2%	1.15[0.87,1.51]
Pre-existing Conditions	1.9%	2.0%	0.95[0.86,1.05]
Developmental disorders			
Late	0.0%	0.0%	1.59[0.62,4.10]
Acute and Persistent		0.0%	
Pre-existing Conditions	0.1%	0.0%	1.70[0.92,3.15]
Diabetes [a]			
Late	0.9%	0.8%	1.20[1.03,1.38]*
Acute and Persistent	0.3%	0.2%	1.96[1.50,2.55]*
Pre-existing Conditions	11.5%	9.3%	1.23[1.18,1.29]*
Digestive congenital anomalies			
Late	0.0%	0.0%	0.25[0.03,1.95]
Pre-existing Conditions	0.0%	0.0%	0.50[0.06,4.28]
Diseases of white blood cells			
Late	0.2%	0.2%	0.92[0.68,1.23]
Acute and Persistent	0.0%	0.0%	1.06[0.52,2.14]
Pre-existing Conditions	0.1%	0.2%	0.69[0.47,1.01]
Diverticulosis and diverticulitis			

Late	0.2%	0.2%	1.26[0.93,1.71]
Acute and Persistent	0.0%	0.0%	0.75[0.30,1.87]
Pre-existing Conditions	0.2%	0.3%	0.62[0.45,0.85]*
Encephalitis (except that caused by tuberculosis or sexually transmitted disease)			
Late	0.0%	0.0%	2.50[0.16,39.97]
Pre-existing Conditions	0.0%		
Epilepsy; convulsions			
Late	0.1%	0.1%	0.71[0.40,1.26]
Acute and Persistent	0.0%	0.0%	0.31[0.09,1.04]
Pre-existing Conditions	0.3%	0.3%	1.05[0.81,1.35]
Esophageal disorders			
Late	1.1%	1.0%	1.14[1.00,1.31]
Acute and Persistent	0.2%	0.2%	1.09[0.82,1.46]
Pre-existing Conditions	3.6%	3.7%	0.98[0.91,1.06]
External cause codes: Cut/pierce			
Late	0.0%	0.0%	0.97[0.41,2.33]
Acute and Persistent	0.0%	0.0%	2.50[0.16,39.97]
Pre-existing Conditions	0.0%	0.0%	2.50[0.16,39.97]
External cause codes: Fire/burn			
Late	0.0%	0.0%	0.83[0.09,8.01]
External cause codes: Firearm			
Late	0.0%	0.0%	1.25[0.11,13.79]
Acute and Persistent		0.0%	
Pre-existing Conditions	0.0%	0.0%	1.87[0.42,8.38]
External cause codes: Machinery			
Late	0.0%	0.0%	2.50[0.16,39.97]
External cause codes: Other specified and classifiable			
Late	0.0%	0.0%	1.33[0.57,3.14]
Acute and Persistent		0.0%	
Pre-existing Conditions	0.0%	0.0%	2.50[0.16,39.97]
External cause codes: Other specified; NEC			
Late		0.0%	
Eye			
Late	0.4%	0.5%	0.86[0.70,1.07]
Acute and Persistent	0.0%	0.0%	0.16[0.04,0.65]*
Pre-existing Conditions	1.8%	2.0%	0.90[0.81,0.99]*
Fetopelvic disproportion; obstruction			
Acute and Persistent		0.0%	
Fluid and electrolyte disorders [a]			
Late	0.4%	0.6%	0.73[0.59,0.90]*

Acute and Persistent	0.2%	0.1%	1.96[1.41,2.74]*
Pre-existing Conditions	0.7%	0.8%	0.82[0.69,0.97]*
General Symptoms and Illness			
Late	0.6%	0.6%	0.95[0.79,1.15]
Acute and Persistent	0.2%	0.2%	1.03[0.75,1.40]
Pre-existing Conditions	5.0%	4.7%	1.05[0.99,1.12]
Genitourinary symptoms and ill-defined conditions [a]			
Late	1.5%	1.2%	1.21[1.07,1.36]*
Acute and Persistent	0.1%	0.1%	1.14[0.75,1.74]
Pre-existing Conditions	2.1%	2.0%	1.04[0.94,1.14]
Gastrointestinal disorders [a]			
Late	1.7%	1.7%	1.00[0.90,1.12]
Acute and Persistent	0.4%	0.4%	0.98[0.79,1.21]
Pre-existing Conditions	3.4%	4.0%	0.84[0.78,0.90]*
Hodgkin`s disease			
Acute and Persistent		0.0%	
Pre-existing Conditions	0.0%	0.0%	0.83[0.33,2.10]
Hypertension			
Late	0.6%	0.6%	1.10[0.92,1.30]
Acute and Persistent	0.3%	0.3%	0.99[0.77,1.27]
Pre-existing Conditions	13.3%	12.5%	1.07[1.03,1.11]*
Immunity disorders			
Late	0.0%	0.1%	0.45[0.20,1.00]
Acute and Persistent	0.0%	0.0%	0.68[0.19,2.44]
Pre-existing Conditions	0.2%	0.2%	0.93[0.67,1.29]
Infectious disease			
Late	1.1%	1.2%	0.98[0.86,1.12]
Acute and Persistent	0.3%	0.2%	1.34[1.04,1.73]*
Pre-existing Conditions	2.3%	2.4%	0.93[0.85,1.01]
Inflammation; infection of eye (except that caused by tuberculosis or sexually transmitted disease)			
Late	0.6%	0.7%	0.93[0.78,1.10]
Acute and Persistent	0.0%	0.0%	1.58[0.77,3.25]
Pre-existing Conditions	0.5%	0.6%	0.85[0.70,1.03]
Inflammatory conditions of male genital organs			
Late	0.1%	0.1%	1.14[0.75,1.74]
Acute and Persistent	0.0%	0.0%	0.71[0.15,3.44]
Pre-existing Conditions	0.0%	0.0%	0.57[0.27,1.23]
Joint disease			
Late	0.9%	0.8%	1.14[0.98,1.31]
Acute and Persistent	0.1%	0.1%	0.77[0.51,1.15]

Pre-existing Conditions	2.7%	3.0%	0.91[0.84,0.99]*
Leukemias			
Late	0.0%	0.0%	0.62[0.13,2.94]
Acute and Persistent	0.0%	0.0%	0.42[0.05,3.46]
Pre-existing Conditions	0.1%	0.1%	1.09[0.69,1.71]
Lung disease due to external agents			
Late	0.0%	0.0%	0.38[0.09,1.70]
Acute and Persistent	0.0%	0.0%	2.50[0.35,17.75]
Pre-existing Conditions	0.0%	0.0%	2.50[0.16,39.97]
Lymphadenitis			
Late	0.2%	0.2%	1.04[0.77,1.40]
Acute and Persistent	0.0%	0.0%	0.59[0.20,1.75]
Pre-existing Conditions	0.1%	0.1%	0.55[0.32,0.96]*
Malaise and fatigue [a]			
Late	1.4%	0.9%	1.60[1.41,1.81]*
Acute and Persistent	0.3%	0.1%	2.89[2.10,3.98]*
Pre-existing Conditions	1.0%	0.9%	1.15[1.00,1.33]*
Malposition; malpresentation			
Late	0.0%	0.0%	1.47[0.67,3.21]
Pre-existing Conditions		0.0%	
Melanomas of skin			
Late	0.0%	0.0%	0.62[0.07,5.59]
Acute and Persistent		0.0%	
Pre-existing Conditions	0.1%	0.1%	1.11[0.62,2.00]
Meningitis (except that caused by tuberculosis or sexually transmitted disease)			
Late	0.0%	0.0%	0.83[0.09,8.01]
Pre-existing Conditions		0.0%	
Mental health [a]			
Late	1.1%	1.2%	0.95[0.83,1.08]
Acute and Persistent	0.2%	0.3%	0.62[0.45,0.86]*
Pre-existing Conditions	4.0%	5.6%	0.71[0.67,0.76]*
Multiple sclerosis			
Late	0.0%	0.0%	0.56[0.12,2.57]
Acute and Persistent	0.0%	0.0%	1.25[0.11,13.79]
Pre-existing Conditions	0.1%	0.1%	0.89[0.59,1.35]
Mycoses			
Late	0.9%	0.8%	1.07[0.92,1.24]
Acute and Persistent	0.0%	0.0%	0.29[0.09,0.95]*
Pre-existing Conditions	1.1%	0.9%	1.18[1.03,1.35]*
Nausea and vomiting [a]			
Late	0.7%	0.7%	0.95[0.80,1.12]

Acute and Persistent	0.1%	0.1%	0.81[0.49,1.32]
Pre-existing Conditions	0.6%	0.7%	0.84[0.71,1.00]
Neuropathy			
Late	0.3%	0.3%	1.06[0.82,1.38]
Acute and Persistent	0.0%	0.0%	0.44[0.13,1.51]
Pre-existing Conditions	0.3%	0.3%	0.99[0.76,1.29]
Nonmalignant breast conditions			
Late	0.6%	0.5%	1.08[0.89,1.30]
Acute and Persistent	0.0%	0.1%	0.21[0.09,0.48]*
Pre-existing Conditions	0.4%	0.5%	0.93[0.76,1.15]
Nonspecific chest pain [a]			
Late	1.7%	1.2%	1.39[1.24,1.55]*
Acute and Persistent	0.4%	0.2%	2.39[1.85,3.10]*
Pre-existing Conditions	1.8%	1.5%	1.25[1.12,1.39]*
OB-related trauma to perineum and vulva			
Late	0.0%	0.0%	1.67[0.47,5.91]
Acute and Persistent		0.0%	
Pre-existing Conditions	0.0%	0.0%	5.00[0.45,55.14]
Open wounds of extremities			
Late	0.3%	0.3%	0.92[0.72,1.17]
Acute and Persistent	0.0%	0.0%	0.75[0.30,1.87]
Pre-existing Conditions	0.1%	0.1%	0.68[0.41,1.11]
Other and unspecified benign neoplasm			
Late	1.0%	1.0%	0.98[0.86,1.13]
Acute and Persistent	0.0%	0.2%	0.29[0.17,0.50]*
Pre-existing Conditions	1.2%	1.5%	0.82[0.73,0.93]*
Other circulatory disease			
Late	1.0%	1.0%	1.01[0.88,1.16]
Acute and Persistent	0.1%	0.2%	0.72[0.50,1.05]
Pre-existing Conditions	1.2%	1.4%	0.82[0.73,0.93]*
Other ear and sense organ disorders			
Late	0.1%	0.2%	0.79[0.55,1.13]
Acute and Persistent	0.0%	0.0%	0.42[0.09,1.86]
Pre-existing Conditions	0.1%	0.1%	0.86[0.58,1.26]
Other endocrine disorders			
Late	0.3%	0.3%	0.83[0.64,1.07]
Acute and Persistent	0.1%	0.0%	1.25[0.70,2.24]
Pre-existing Conditions	0.6%	0.7%	0.94[0.79,1.12]
Other eye disorders			
Late	1.2%	1.1%	1.06[0.94,1.21]
Acute and Persistent	0.0%	0.1%	0.12[0.03,0.50]*

Pre-existing Conditions	1.2%	1.5%	0.78[0.69,0.88]*
Other hematologic conditions			
Late	0.2%	0.2%	0.96[0.68,1.37]
Acute and Persistent	0.0%	0.0%	0.91[0.29,2.85]
Pre-existing Conditions	0.1%	0.1%	0.67[0.40,1.11]
Other infections; including parasitic			
Late	0.1%	0.0%	4.57[2.75,7.59]*
Acute and Persistent	0.0%	0.0%	1.56[0.51,4.78]
Pre-existing Conditions	0.0%	0.0%	1.67[0.28,9.97]
Other liver disease			
Late	0.2%	0.2%	1.10[0.82,1.47]
Acute and Persistent	0.1%	0.1%	1.01[0.56,1.85]
Pre-existing Conditions	0.2%	0.2%	0.84[0.62,1.16]
Other liver diseases			
Late	0.7%	0.5%	1.24[1.04,1.48]*
Acute and Persistent	0.1%	0.1%	1.20[0.82,1.76]
Pre-existing Conditions	1.1%	1.2%	0.94[0.83,1.07]
Other lower respiratory disease[a]			
Late	1.1%	1.2%	0.92[0.80,1.04]
Acute and Persistent	1.2%	0.5%	2.51[2.15,2.92]*
Pre-existing Conditions	12.2%	10.0%	1.21[1.16,1.26]*
Other nervous system disorders [a]			
Late	0.7%	0.7%	1.04[0.89,1.22]
Acute and Persistent	0.1%	0.1%	0.97[0.64,1.49]
Pre-existing Conditions	1.3%	1.5%	0.87[0.77,0.98]*
Other nutritional; endocrine; and metabolic disorders [a]			
Late	0.5%	0.4%	1.14[0.93,1.40]
Acute and Persistent	0.0%	0.1%	0.54[0.30,0.99]*
Pre-existing Conditions	0.4%	0.4%	0.82[0.66,1.03]
Otitis media and related conditions			
Late	0.3%	0.3%	0.88[0.68,1.14]
Acute and Persistent	0.0%	0.0%	0.96[0.34,2.70]
Pre-existing Conditions	0.2%	0.2%	1.03[0.74,1.44]
Paralysis			
Late	0.0%	0.0%	1.11[0.48,2.56]
Acute and Persistent	0.0%	0.0%	0.31[0.04,2.50]
Pre-existing Conditions	0.1%	0.1%	0.97[0.55,1.69]
Parkinson`s disease			
Late	0.0%	0.0%	0.23[0.03,1.76]
Acute and Persistent	0.0%	0.0%	2.50[0.35,17.75]
Pre-existing Conditions	0.1%	0.1%	0.71[0.40,1.26]

Peripheral and visceral atherosclerosis			
Late	0.4%	0.4%	0.95[0.76,1.18]
Acute and Persistent	0.1%	0.1%	0.97[0.66,1.44]
Pre-existing Conditions	1.9%	2.0%	0.93[0.85,1.03]
Peritonitis and intestinal abscess			
Late	0.0%	0.0%	0.29[0.07,1.27]
Acute and Persistent	0.0%	0.0%	0.25[0.03,1.95]
Pre-existing Conditions	0.0%	0.0%	1.00[0.19,5.15]
Personality disorders			
Late	0.0%	0.0%	0.83[0.17,4.13]
Acute and Persistent	0.0%	0.0%	0.62[0.07,5.59]
Pre-existing Conditions	0.0%	0.0%	0.65[0.27,1.60]
Prolonged pregnancy			
Late		0.0%	
Acute and Persistent	0.0%		
Regional enteritis and ulcerative colitis			
Late	0.0%	0.0%	0.43[0.15,1.26]
Acute and Persistent	0.0%	0.0%	0.21[0.03,1.60]
Pre-existing Conditions	0.2%	0.3%	0.71[0.54,0.94]*
Renal			
Late	0.4%	0.5%	0.83[0.67,1.02]
Acute and Persistent	0.2%	0.2%	1.21[0.88,1.66]
Pre-existing Conditions	2.4%	2.6%	0.94[0.86,1.02]
Respiratory failure; insufficiency; arrest (adult) [a]			
Late	0.1%	0.1%	1.14[0.73,1.80]
Acute and Persistent	0.7%	0.0%	22.95[14.78,35.64]*
Pre-existing Conditions	0.3%	0.2%	1.72[1.29,2.29]*
Rheumatoid arthritis and related disease			
Late	0.0%	0.0%	0.53[0.23,1.20]
Acute and Persistent		0.0%	
Pre-existing Conditions	0.4%	0.4%	0.89[0.72,1.11]
Shock			
Late	0.0%	0.0%	0.30[0.09,0.99]*
Acute and Persistent	0.0%		
Pre-existing Conditions	0.0%	0.0%	0.50[0.06,4.28]
Short gestation; low birth weight; and fetal growth retardation			
Late		0.0%	
Sickle cell anemia			
Late	0.0%	0.0%	1.43[0.70,2.90]
Acute and Persistent	0.0%	0.0%	7.50[0.78,72.10]

Pre-existing Conditions	0.2%	0.2%	1.23[0.91,1.66]
Skin Sensitivity			
Late	0.7%	0.6%	1.13[0.96,1.33]
Acute and Persistent	0.1%	0.0%	1.79[0.92,3.46]
Pre-existing Conditions	0.3%	0.3%	0.92[0.71,1.18]
Spinal cord injury			
Late		0.0%	
Acute and Persistent	0.0%		
Pre-existing Conditions	0.0%	0.0%	1.07[0.28,4.14]
Systemic lupus erythematosus and connective tissue disorders			
Late	0.0%	0.0%	0.50[0.17,1.46]
Acute and Persistent	0.0%	0.0%	0.36[0.04,2.90]
Pre-existing Conditions	0.2%	0.4%	0.64[0.49,0.83]*
Thyroid disorders			
Late	0.4%	0.3%	1.18[0.94,1.48]
Acute and Persistent	0.1%	0.1%	0.74[0.48,1.16]
Pre-existing Conditions	2.8%	2.9%	0.96[0.88,1.04]
Urinary tract infections			
Late	0.8%	0.8%	1.01[0.87,1.18]
Acute and Persistent	0.1%	0.1%	0.69[0.40,1.20]
Pre-existing Conditions	0.9%	1.0%	0.94[0.81,1.08]
Vascular Disease & CVD			
Late	0.6%	0.5%	1.08[0.90,1.30]
Acute and Persistent	0.3%	0.3%	0.90[0.69,1.17]
Pre-existing Conditions	2.9%	3.1%	0.96[0.89,1.04]

* Represents risk ratios with $p<0.05$; [a] PASC Related Conditions; Time periods were defined as follows: Late: 30-120 days post COVID test date; Acute and Acute and Persistent 0-30 days post COVID test date and persisted 30-120 days; Pre-existing conditions: 4 years prior to COVID test date

Supplementary Table 2: CCS Categories Deleted from Analysis

CCS CATEGORY	CCS CATEGORY DESCRIPTION
143	Abdominal hernia
208	Acquired foot deformities
60	Acute posthemorrhagic anemia
255	Administrative/social admission
2617	Adverse effects of medical drugs
253	Allergic reactions
129	Aspiration pneumonitis; food/vomitus
46	Benign neoplasm of uterus
89	Blindness and vision defects

240	Burns
160	Calculus of urinary tract
107	Cardiac arrest and ventricular fibrillation
86	Cataract
237	Complication of device; implant or graft
238	Complications of surgical procedures or medical care
105	Conduction disorders
176	Contraceptive and procreative management
234	Crushing injury or internal injury
186	Diabetes or abnormal glucose tolerance complicating pregnancy; childbirth; or the puerperium
53	Disorders of lipid metabolism
136	Disorders of teeth and jaw
655	Disorders usually diagnosed in infancy childhood or adolescence
184	Early or threatened labor
180	Ectopic pregnancy
169	Endometriosis
2603	External cause codes: Fall
2607	External cause codes: Motor vehicle traffic (MVT)
2611	External cause codes: Natural/environment
2612	External cause codes: Overexertion
2608	External cause codes: Pedal cyclist; not MVT
2609	External cause codes: Pedestrian; not MVT
2621	External cause codes: Place of occurrence
2614	External cause codes: Struck by; against
2610	External cause codes: Transport; not MVT
2620	External cause codes: Unspecified
174	Female infertility
190	Fetal distress and abnormal forces of labor
230	Fracture of lower limb
226	Fracture of neck of femur (hip)
229	Fracture of upper limb
215	Genitourinary congenital anomalies
182	Hemorrhage during pregnancy; abruptio placenta; placenta previa
120	Hemorrhoids
164	Hyperplasia of prostate
183	Hypertension complicating pregnancy; childbirth and the puerperium
10	Immunizations and screening for infectious disease
178	Induced abortion

168	Inflammatory diseases of female pelvic organs
145	Intestinal obstruction without hernia
233	Intracranial injury
225	Joint disorders and dislocations; trauma-related
113	Late effects of cerebrovascular disease
218	Liveborn
45	Maintenance chemotherapy; radiotherapy
256	Medical examination/evaluation
173	Menopausal disorders
171	Menstrual disorders
44	Neoplasms of unspecified nature or uncertain behavior
216	Nervous system congenital anomalies
52	Nutritional deficiencies
235	Open wounds of head; neck; and trunk
206	Osteoporosis
209	Other acquired deformities
257	Other aftercare
195	Other complications of birth; puerperium affecting management of mother
181	Other complications of pregnancy
217	Other congenital anomalies
211	Other connective tissue disease
175	Other female genital disorders
231	Other fractures
81	Other hereditary and degenerative nervous system conditions
244	Other injuries and conditions due to external causes
166	Other male genital disorders
204	Other non-traumatic joint disorders
224	Other perinatal conditions
196	Other pregnancy and delivery including normal
258	Other screening for suspected conditions (not mental disorders or infectious disease)
200	Other skin disorders
172	Ovarian cyst
207	Pathological fracture
243	Poisoning by nonmedicinal substances
242	Poisoning by other medications and drugs
241	Poisoning by psychotropic agents
191	Polyhydramnios and other problems of amniotic cavity
189	Previous C-section
170	Prolapse of female genital organs

254	Rehabilitation care; fitting of prostheses; and adjustment of devices
259	Residual codes; unclassified
663	Screening and history of mental health and substance abuse codes
42	Secondary malignancies
9	Sexually transmitted infections (not HIV or hepatitis)
228	Skull and face fractures
205	Spondylosis; intervertebral disc disorders; other back problems
177	Spontaneous abortion
232	Sprains and strains
239	Superficial injury; contusion
192	Umbilical cord complication

CCS Categories deleted based on clinical determination that they were not relevant to COVID-19

Supplementary Table 3: Specific ICD Diagnoses Deleted from Analysis

CCS CATEGORY	CCS CATEGORY DESCRIPTION	ICD-10 CM CODE	ICD 10 CM CODE DESCRIPTION
163	Genitourinary symptoms and ill-defined conditions	R34	Anuria and oliguria
163	Genitourinary symptoms and ill-defined conditions	R3121	Asymptomatic microscopic hematuria
163	Genitourinary symptoms and ill-defined conditions	R8271	Bacteriuria
163	Genitourinary symptoms and ill-defined conditions	R3982	Chronic bladder pain
163	Genitourinary symptoms and ill-defined conditions	N3945	Continuous leakage
163	Genitourinary symptoms and ill-defined conditions	N399	Disorder of urinary system, unspecified
163	Genitourinary symptoms and ill-defined conditions	Z466	Encounter for fitting and adjustment of urinary device
163	Genitourinary symptoms and ill-defined conditions	R392	Extrarenal uremia
163	Genitourinary symptoms and ill-defined conditions	R3914	Feeling of incomplete bladder emptying
163	Genitourinary symptoms and ill-defined conditions	R3911	Hesitancy of micturition
163	Genitourinary symptoms and ill-defined conditions	N3946	Mixed incontinence
163	Genitourinary symptoms and ill-defined conditions	R351	Nocturia
163	Genitourinary symptoms and ill-defined conditions	N3944	Nocturnal enuresis
163	Genitourinary symptoms and ill-defined conditions	N139	Obstructive and reflux uropathy, unspecified
163	Genitourinary symptoms and ill-defined conditions	R82998	Other abnormal findings in urine
163	Genitourinary symptoms and ill-defined conditions	Z936	Other artificial openings of urinary tract status
163	Genitourinary symptoms and ill-defined conditions	R39198	Other difficulties with micturition
163	Genitourinary symptoms and ill-defined conditions	R358	Other polyuria
163	Genitourinary symptoms and ill-defined conditions	R338	Other retention of urine
163	Genitourinary symptoms and ill-defined conditions	R3989	Other symptoms and signs involving the genitourinary system
163	Genitourinary symptoms and ill-defined conditions	Z87448	Personal history of other diseases of urinary system
163	Genitourinary symptoms and ill-defined conditions	Z87440	Personal history of urinary (tract) infections

163	Genitourinary symptoms and ill-defined conditions	R3912	Poor urinary stream
163	Genitourinary symptoms and ill-defined conditions	N39492	Postural (urinary) incontinence
163	Genitourinary symptoms and ill-defined conditions	N3943	Post-void dribbling
163	Genitourinary symptoms and ill-defined conditions	Z960	Presence of urogenital implants
163	Genitourinary symptoms and ill-defined conditions	R339	Retention of urine, unspecified
163	Genitourinary symptoms and ill-defined conditions	R3913	Splitting of urinary stream
163	Genitourinary symptoms and ill-defined conditions	N393	Stress incontinence (female) (male)
163	Genitourinary symptoms and ill-defined conditions	R399	Unspecified symptoms and signs involving the genitourinary system
163	Genitourinary symptoms and ill-defined conditions	R8290	Unspecified abnormal findings in urine
163	Genitourinary symptoms and ill-defined conditions	Z9350	Unspecified cystostomy status
163	Genitourinary symptoms and ill-defined conditions	R32	Unspecified urinary incontinence
163	Genitourinary symptoms and ill-defined conditions	R369	Urethral discharge, unspecified
163	Genitourinary symptoms and ill-defined conditions	N369	Urethral disorder, unspecified
163	Genitourinary symptoms and ill-defined conditions	N3941	Urge incontinence
252	Malaise and fatigue	R530	Neoplastic (malignant) related fatigue
94	Other ear and sense organ disorders	H6002	Abscess of left external ear
94	Other ear and sense organ disorders	H60543	Acute eczematoid otitis externa, bilateral
94	Other ear and sense organ disorders	H60542	Acute eczematoid otitis externa, left ear
94	Other ear and sense organ disorders	H60541	Acute eczematoid otitis externa, right ear
94	Other ear and sense organ disorders	H7102	Cholesteatoma of attic, left ear
94	Other ear and sense organ disorders	H7111	Cholesteatoma of tympanum, right ear
94	Other ear and sense organ disorders	H7311	Chronic myringitis, right ear
94	Other ear and sense organ disorders	Z9621	Cochlear implant status
94	Other ear and sense organ disorders	H933X2	Disorders of left acoustic nerve

94	Other ear and sense organ disorders	H933X1	Disorders of right acoustic nerve
94	Other ear and sense organ disorders	Z461	Encounter for fitting and adjustment of hearing aid
94	Other ear and sense organ disorders	H93231	Hyperacusis, right ear
94	Other ear and sense organ disorders	H6123	Impacted cerumen, bilateral
94	Other ear and sense organ disorders	H6122	Impacted cerumen, left ear
94	Other ear and sense organ disorders	H6121	Impacted cerumen, right ear
94	Other ear and sense organ disorders	H6120	Impacted cerumen, unspecified ear
94	Other ear and sense organ disorders	H9071	Mix conductive/sensorineural hearing loss, unilateral right ear, with unrestricted hearing contralateral side
94	Other ear and sense organ disorders	Z9622	Myringotomy tube(s) status
94	Other ear and sense organ disorders	H833X3	Noise effects on inner ear, bilateral
94	Other ear and sense organ disorders	H9203	Otalgia, bilateral
94	Other ear and sense organ disorders	H9202	Otalgia, left ear
94	Other ear and sense organ disorders	H9201	Otalgia, right ear
94	Other ear and sense organ disorders	H9209	Otalgia, unspecified ear
94	Other ear and sense organ disorders	H938X9	Other specified disorders of ear, unspecified ear
94	Other ear and sense organ disorders	H938X2	Other specified disorders of left ear
94	Other ear and sense organ disorders	H61892	Other specified disorders of left external ear
94	Other ear and sense organ disorders	H748X2	Other specified disorders of left middle ear and mastoid
94	Other ear and sense organ disorders	H938X1	Other specified disorders of right ear
94	Other ear and sense organ disorders	H748X1	Other specified disorders of right middle ear and mastoid
94	Other ear and sense organ disorders	H6240	Otitis externa in other diseases classified elsewhere, unspecified ear
94	Other ear and sense organ disorders	H9221	Otorrhagia, right ear
94	Other ear and sense organ disorders	H9212	Otorrhea, left ear

94	Other ear and sense organ disorders	H9211	Otorrhea, right ear
94	Other ear and sense organ disorders	H93A3	Pulsatile tinnitus, bilateral
94	Other ear and sense organ disorders	H93A2	Pulsatile tinnitus, left ear
94	Other ear and sense organ disorders	H93A1	Pulsatile tinnitus, right ear
94	Other ear and sense organ disorders	H903	Sensorineural hearing loss, bilateral
94	Other ear and sense organ disorders	H90A22	Sensorineural hearing loss, unilateral, l ear, with restricted hear contralateral side
94	Other ear and sense organ disorders	H9313	Tinnitus, bilateral
94	Other ear and sense organ disorders	H9312	Tinnitus, left ear
94	Other ear and sense organ disorders	H9311	Tinnitus, right ear
94	Other ear and sense organ disorders	H9319	Tinnitus, unspecified ear
94	Other ear and sense organ disorders	H60503	Unspecified acute noninfective otitis externa, bilateral
94	Other ear and sense organ disorders	H60502	Unspecified acute noninfective otitis externa, left ear
94	Other ear and sense organ disorders	H60501	Unspecified acute noninfective otitis externa, right ear
94	Other ear and sense organ disorders	H7192	Unspecified cholesteatoma, left ear
94	Other ear and sense organ disorders	H6063	Unspecified chronic otitis externa, bilateral
94	Other ear and sense organ disorders	H6062	Unspecified chronic otitis externa, left ear
94	Other ear and sense organ disorders	H9393	Unspecified disorder of ear, bilateral
94	Other ear and sense organ disorders	H9390	Unspecified disorder of ear, unspecified ear
94	Other ear and sense organ disorders	H9392	Unspecified disorder of left ear
94	Other ear and sense organ disorders	H9391	Unspecified disorder of right ear
94	Other ear and sense organ disorders	H7491	Unspecified disorder of right middle ear and mastoid
94	Other ear and sense organ disorders	H9193	Unspecified hearing loss, bilateral
94	Other ear and sense organ disorders	H9191	Unspecified hearing loss, right ear
94	Other ear and sense organ disorders	H9190	Unspecified hearing loss, unspecified ear

94	Other ear and sense organ disorders	H6093	Unspecified otitis externa, bilateral
94	Other ear and sense organ disorders	H6092	Unspecified otitis externa, left ear
94	Other ear and sense organ disorders	H6091	Unspecified otitis externa, right ear
94	Other ear and sense organ disorders	H61001	Unspecified perichondritis of right external ear
91	Other eye disorders	H44513	Absolute glaucoma, bilateral
91	Other eye disorders	H44512	Absolute glaucoma, left eye
91	Other eye disorders	H04553	Acquired stenosis of bilateral nasolacrimal duct
91	Other eye disorders	H04552	Acquired stenosis of left nasolacrimal duct
91	Other eye disorders	H04551	Acquired stenosis of right nasolacrimal duct
91	Other eye disorders	H5005	Alternating esotropia
91	Other eye disorders	H5015	Alternating exotropia
91	Other eye disorders	H2702	Aphakia, left eye
91	Other eye disorders	H2701	Aphakia, right eye
91	Other eye disorders	H18413	Arcus senilis, bilateral
91	Other eye disorders	H44522	Atrophy of globe, left eye
91	Other eye disorders	H0236	Blepharochalasis left eye, unspecified eyelid
91	Other eye disorders	H0234	Blepharochalasis left upper eyelid
91	Other eye disorders	H0233	Blepharochalasis right eye, unspecified eyelid
91	Other eye disorders	H57813	Brow ptosis, bilateral
91	Other eye disorders	H57811	Brow ptosis, right
91	Other eye disorders	H1813	Bullous keratopathy, bilateral
91	Other eye disorders	Z9842	Cataract extraction status, left eye
91	Other eye disorders	Z9841	Cataract extraction status, right eye
91	Other eye disorders	Z9849	Cataract extraction status, unspecified eye
91	Other eye disorders	H16011	Central corneal ulcer, right eye
91	Other eye disorders	H0016	Chalazion left eye, unspecified eyelid
91	Other eye disorders	H0015	Chalazion left lower eyelid
91	Other eye disorders	H0014	Chalazion left upper eyelid
91	Other eye disorders	H0012	Chalazion right lower eyelid
91	Other eye disorders	H0011	Chalazion right upper eyelid
91	Other eye disorders	H32	Chorioretinal disorders in diseases classified elsewhere
91	Other eye disorders	H11442	Conjunctival cysts, left eye
91	Other eye disorders	H11441	Conjunctival cysts, right eye
91	Other eye disorders	H11433	Conjunctival hyperemia, bilateral
91	Other eye disorders	H11432	Conjunctival hyperemia, left eye
91	Other eye disorders	H11133	Conjunctival pigmentations, bilateral
91	Other eye disorders	H11823	Conjunctivochalasis, bilateral
91	Other eye disorders	H5111	Convergence insufficiency

91	Other eye disorders	H18823	Corneal disorder due to contact lens, bilateral
91	Other eye disorders	H18821	Corneal disorder due to contact lens, right eye
91	Other eye disorders	H18713	Corneal ectasia, bilateral
91	Other eye disorders	Z947	Corneal transplant status
91	Other eye disorders	H4323	Crystalline deposits in vitreous body, bilateral
91	Other eye disorders	H4322	Crystalline deposits in vitreous body, left eye
91	Other eye disorders	H4321	Crystalline deposits in vitreous body, right eye
91	Other eye disorders	H02825	Cysts of left lower eyelid
91	Other eye disorders	H02823	Cysts of right eye, unspecified eyelid
91	Other eye disorders	H05332	Deformity of left orbit due to trauma or surgery
91	Other eye disorders	H05331	Deformity of right orbit due to trauma or surgery
91	Other eye disorders	H21233	Degeneration of iris (pigmentary), bilateral
91	Other eye disorders	H442E3	Degenerative myopia with other maculopathy, bilateral eye
91	Other eye disorders	H4423	Degenerative myopia, bilateral
91	Other eye disorders	H02836	Dermatochalasis of left eye, unspecified eyelid
91	Other eye disorders	H02834	Dermatochalasis of left upper eyelid
91	Other eye disorders	H02833	Dermatochalasis of right eye, unspecified eyelid
91	Other eye disorders	H02831	Dermatochalasis of right upper eyelid
91	Other eye disorders	H11031	Double pterygium of right eye
91	Other eye disorders	H04122	Dry eye syndrome of left lacrimal gland
91	Other eye disorders	H50812	Duane's syndrome, left eye
91	Other eye disorders	H05223	Edema of bilateral orbit
91	Other eye disorders	H02846	Edema of left eye, unspecified eyelid
91	Other eye disorders	H02845	Edema of left lower eyelid
91	Other eye disorders	H02844	Edema of left upper eyelid
91	Other eye disorders	H02843	Edema of right eye, unspecified eyelid
91	Other eye disorders	H05221	Edema of right orbit
91	Other eye disorders	H02841	Edema of right upper eyelid
91	Other eye disorders	Z460	Encounter for fit/adjustment of spectacles and contact lenses
91	Other eye disorders	H1851	Endothelial corneal dystrophy
91	Other eye disorders	H04212	Epiphora due to excess lacrimation, left lacrimal gland
91	Other eye disorders	H1852	Epithelial (juvenile) corneal dystrophy
91	Other eye disorders	H5052	Exophoria

91	Other eye disorders	H2181	Floppy iris syndrome
91	Other eye disorders	H2102	Hyphema, left eye
91	Other eye disorders	H2101	Hyphema, right eye
91	Other eye disorders	H5034	Intermittent alternating exotropia
91	Other eye disorders	H5123	Internuclear ophthalmoplegia, bilateral
91	Other eye disorders	H47012	Ischemic optic neuropathy, left eye
91	Other eye disorders	H47011	Ischemic optic neuropathy, right eye
91	Other eye disorders	H18613	Keratoconus, stable, bilateral
91	Other eye disorders	H18603	Keratoconus, unspecified, bilateral
91	Other eye disorders	H18602	Keratoconus, unspecified, left eye
91	Other eye disorders	H31013	Macula scars of posterior pole (post-traumatic), bilateral
91	Other eye disorders	H31012	Macula scars of posterior pole (post-traumatic), left eye
91	Other eye disorders	H31011	Macula scars of posterior pole (post-traumatic), right eye
91	Other eye disorders	H02726	Madarosis of left eye, unspecified eyelid and periocular area
91	Other eye disorders	H02723	Madarosis of right eye, unspecified eyelid and periocular area
91	Other eye disorders	H16041	Marginal corneal ulcer, right eye
91	Other eye disorders	H02413	Mechanical ptosis of bilateral eyelids
91	Other eye disorders	H02412	Mechanical ptosis of left eyelid
91	Other eye disorders	H02886	Meibomian gland dysfunction of left eye, unspecified eyelid
91	Other eye disorders	H02883	Meibomian gland dysfunction of right eye, unspecified eyelid
91	Other eye disorders	H18453	Nodular corneal degeneration, bilateral
91	Other eye disorders	H18451	Nodular corneal degeneration, right eye
91	Other eye disorders	H47093	Other disorders of optic nerve, NEC, bilateral
91	Other eye disorders	H47091	Other disorders of optic nerve, NEC, right eye
91	Other eye disorders	H5709	Other anomalies of pupillary function
91	Other eye disorders	H31093	Other chorioretinal scars, bilateral
91	Other eye disorders	H31092	Other chorioretinal scars, left eye
91	Other eye disorders	H31091	Other chorioretinal scars, right eye
91	Other eye disorders	H0589	Other disorders of orbit
91	Other eye disorders	H5589	Other irregular eye movements
91	Other eye disorders	H318	Other specified disorders of choroid
91	Other eye disorders	H18899	Other specified disorders of cornea, unspecified eye
91	Other eye disorders	H0289	Other specified disorders of eyelid
91	Other eye disorders	H0419	Other specified disorders of lacrimal gland
91	Other eye disorders	H211X2	Other vascular disorders of iris and ciliary body, left eye

91	Other eye disorders	H43393	Other vitreous opacities, bilateral
91	Other eye disorders	H43392	Other vitreous opacities, left eye
91	Other eye disorders	H43391	Other vitreous opacities, right eye
91	Other eye disorders	H4711	Papilledema associated with increased intracranial pressure
91	Other eye disorders	H17823	Peripheral opacity of cornea, bilateral
91	Other eye disorders	H11053	Peripheral pterygium, progressive, bilateral
91	Other eye disorders	H11052	Peripheral pterygium, progressive, left eye
91	Other eye disorders	H11043	Peripheral pterygium, stationary, bilateral
91	Other eye disorders	H11153	Pinguecula, bilateral
91	Other eye disorders	H11152	Pinguecula, left eye
91	Other eye disorders	H11151	Pinguecula, right eye
91	Other eye disorders	H10811	Pingueculitis, right eye
91	Other eye disorders	H21543	Posterior synechiae (iris), bilateral
91	Other eye disorders	Z970	Presence of artificial eye
91	Other eye disorders	H21562	Pupillary abnormality, left eye
91	Other eye disorders	H21552	Recession of chamber angle, left eye
91	Other eye disorders	H18832	Recurrent erosion of cornea, left eye
91	Other eye disorders	H18831	Recurrent erosion of cornea, right eye
91	Other eye disorders	H11062	Recurrent pterygium of left eye
91	Other eye disorders	H11242	Scarring of conjunctiva, left eye
91	Other eye disorders	H02036	Senile entropion of left eye, unspecified eyelid
91	Other eye disorders	H02033	Senile entropion of right eye, unspecified eyelid
91	Other eye disorders	H4922	Sixth [abducent] nerve palsy, left eye
91	Other eye disorders	H4921	Sixth [abducent] nerve palsy, right eye
91	Other eye disorders	H31021	Solar retinopathy, right eye
91	Other eye disorders	H02046	Spastic entropion of left eye, unspecified eyelid
91	Other eye disorders	H15831	Staphyloma posticum, right eye
91	Other eye disorders	H04563	Stenosis of bilateral lacrimal punctum
91	Other eye disorders	H04542	Stenosis of left lacrimal canaliculi
91	Other eye disorders	H11233	Symblepharon, bilateral
91	Other eye disorders	H4901	Third [oculomotor] nerve palsy, right eye
91	Other eye disorders	H4900	Third [oculomotor] nerve palsy, unspecified eye
91	Other eye disorders	H57051	Tonic pupil, right eye
91	Other eye disorders	H02056	Trichiasis without entropion left eye, unspecified eyelid
91	Other eye disorders	H02053	Trichiasis without entropion right eye, unspecified eyelid
91	Other eye disorders	H31003	Unspecified chorioretinal scars, bilateral
91	Other eye disorders	H31001	Unspecified chorioretinal scars, right eye
91	Other eye disorders	H31402	Unspecified choroidal detachment, left eye
91	Other eye disorders	H1820	Unspecified corneal edema

91	Other eye disorders	H16401	Unspecified corneal neovascularization, right eye
91	Other eye disorders	H179	Unspecified corneal scar and opacity
91	Other eye disorders	H16003	Unspecified corneal ulcer, bilateral
91	Other eye disorders	H16002	Unspecified corneal ulcer, left eye
91	Other eye disorders	H16001	Unspecified corneal ulcer, right eye
91	Other eye disorders	H119	Unspecified disorder of conjunctiva
91	Other eye disorders	H189	Unspecified disorder of cornea
91	Other eye disorders	H029	Unspecified disorder of eyelid
91	Other eye disorders	H219	Unspecified disorder of iris and ciliary body
91	Other eye disorders	H02106	Unspecified ectropion of left eye, unspecified eyelid
91	Other eye disorders	H02103	Unspecified ectropion of right eye, unspecified eyelid
91	Other eye disorders	H05402	Unspecified enophthalmos, left eye
91	Other eye disorders	H02006	Unspecified entropion of left eye, unspecified eyelid
91	Other eye disorders	H02005	Unspecified entropion of left lower eyelid
91	Other eye disorders	H02003	Unspecified entropion of right eye, unspecified eyelid
91	Other eye disorders	H02002	Unspecified entropion of right lower eyelid
91	Other eye disorders	H04203	Unspecified epiphora, bilateral
91	Other eye disorders	H04202	Unspecified epiphora, left side
91	Other eye disorders	H04201	Unspecified epiphora, right side
91	Other eye disorders	H5000	Unspecified esotropia
91	Other eye disorders	H0520	Unspecified exophthalmos
91	Other eye disorders	H5010	Unspecified exotropia
91	Other eye disorders	H5030	Unspecified intermittent heterotropia
91	Other eye disorders	H02206	Unspecified lagophthalmos left eye, unspecified eyelid
91	Other eye disorders	H02203	Unspecified lagophthalmos right eye, unspecified eyelid
91	Other eye disorders	H5500	Unspecified nystagmus
91	Other eye disorders	H4720	Unspecified optic atrophy
91	Other eye disorders	H11003	Unspecified pterygium of eye, bilateral
91	Other eye disorders	H11002	Unspecified pterygium of left eye
91	Other eye disorders	H11001	Unspecified pterygium of right eye
91	Other eye disorders	H02403	Unspecified ptosis of bilateral eyelids
91	Other eye disorders	H02402	Unspecified ptosis of left eyelid
91	Other eye disorders	H02401	Unspecified ptosis of right eyelid
91	Other eye disorders	H509	Unspecified strabismus
91	Other eye disorders	H5022	Vertical strabismus, left eye
91	Other eye disorders	H5021	Vertical strabismus, right eye
91	Other eye disorders	H43822	Vitreomacular adhesion, left eye
91	Other eye disorders	H43821	Vitreomacular adhesion, right eye
91	Other eye disorders	H43813	Vitreous degeneration, bilateral

91	Other eye disorders	H43812	Vitreous degeneration, left eye
91	Other eye disorders	H43811	Vitreous degeneration, right eye
91	Other eye disorders	H4313	Vitreous hemorrhage, bilateral
91	Other eye disorders	H4312	Vitreous hemorrhage, left eye
91	Other eye disorders	H0266	Xanthelasma of left eye, unspecified eyelid
91	Other eye disorders	H0263	Xanthelasma of right eye, unspecified eyelid
91	Other eye disorders	H0261	Xanthelasma of right upper eyelid
8	Other infections; including parasitic	B889	Infestation, unspecified
8	Other infections; including parasitic	Z8619	Personal history of other infectious and parasitic diseases
8	Other infections; including parasitic	B853	Phthiriasis
8	Other infections; including parasitic	B86	Scabies
8	Other infections; including parasitic	A5903	Trichomonal cystitis and urethritis
8	Other infections; including parasitic	A5901	Trichomonal vulvovaginitis
8	Other infections; including parasitic	A599	Trichomoniasis, unspecified
133	Other lower respiratory disease	R0603	Acute respiratory distress
133	Other lower respiratory disease	R0681	Apnea, not elsewhere classified
133	Other lower respiratory disease	J986	Disorders of diaphragm
133	Other lower respiratory disease	Z942	Lung transplant status
133	Other lower respiratory disease	R0601	Orthopnea
133	Other lower respiratory disease	R918	Other nonspecific abnormal finding of lung field
133	Other lower respiratory disease	Z8709	Personal history of other diseases of the respiratory system
133	Other lower respiratory disease	Z8701	Personal history of pneumonia (recurrent)
133	Other lower respiratory disease	R911	Solitary pulmonary nodule
58	Other nutritional; endocrine; and metabolic disorders	E8801	Alpha-1-antitrypsin deficiency
58	Other nutritional; endocrine; and metabolic disorders	E859	Amyloidosis, unspecified
58	Other nutritional; endocrine; and metabolic disorders	Z6854	BMI pediatric, greater than or equal to 95% for age
58	Other nutritional; endocrine; and metabolic disorders	Z6825	Body mass index (BMI) 25.0-25.9, adult

58	Other nutritional; endocrine; and metabolic disorders	Z6826	Body mass index (BMI) 26.0-26.9, adult
58	Other nutritional; endocrine; and metabolic disorders	Z6827	Body mass index (BMI) 27.0-27.9, adult
58	Other nutritional; endocrine; and metabolic disorders	Z6828	Body mass index (BMI) 28.0-28.9, adult
58	Other nutritional; endocrine; and metabolic disorders	Z6829	Body mass index (BMI) 29.0-29.9, adult
58	Other nutritional; endocrine; and metabolic disorders	Z6830	Body mass index (BMI) 30.0-30.9, adult
58	Other nutritional; endocrine; and metabolic disorders	Z6831	Body mass index (BMI) 31.0-31.9, adult
58	Other nutritional; endocrine; and metabolic disorders	Z6832	Body mass index (BMI) 32.0-32.9, adult
58	Other nutritional; endocrine; and metabolic disorders	Z6833	Body mass index (BMI) 33.0-33.9, adult
58	Other nutritional; endocrine; and metabolic disorders	Z6834	Body mass index (BMI) 34.0-34.9, adult
58	Other nutritional; endocrine; and metabolic disorders	Z6835	Body mass index (BMI) 35.0-35.9, adult
58	Other nutritional; endocrine; and metabolic disorders	Z6836	Body mass index (BMI) 36.0-36.9, adult
58	Other nutritional; endocrine; and metabolic disorders	Z6837	Body mass index (BMI) 37.0-37.9, adult
58	Other nutritional; endocrine; and metabolic disorders	Z6838	Body mass index (BMI) 38.0-38.9, adult
58	Other nutritional; endocrine; and metabolic disorders	Z6839	Body mass index (BMI) 39.0-39.9, adult
58	Other nutritional; endocrine; and metabolic disorders	Z6841	Body mass index (BMI) 40.0-44.9, adult
58	Other nutritional; endocrine; and metabolic disorders	Z6842	Body mass index (BMI) 45.0-49.9, adult
58	Other nutritional; endocrine; and metabolic disorders	Z6843	Body mass index (BMI) 50-59.9, adult
58	Other nutritional; endocrine; and metabolic disorders	Z6844	Body mass index (BMI) 60.0-69.9, adult
58	Other nutritional; endocrine; and metabolic disorders	Z6845	Body mass index (BMI) 70 or greater, adult
58	Other nutritional; endocrine; and metabolic disorders	E700	Classical phenylketonuria
58	Other nutritional; endocrine; and metabolic disorders	E7870	Disorder of bile acid and cholesterol metabolism, unspecified
58	Other nutritional; endocrine; and metabolic disorders	E749	Disorder of carbohydrate metabolism, unspecified
58	Other nutritional; endocrine; and metabolic disorders	E8300	Disorder of copper metabolism, unspecified
58	Other nutritional; endocrine; and metabolic disorders	E789	Disorder of lipoprotein metabolism, unspecified

58	Other nutritional; endocrine; and metabolic disorders	E8330	Disorder of phosphorus metabolism, unspecified
58	Other nutritional; endocrine; and metabolic disorders	E7220	Disorder of urea cycle metabolism, unspecified
58	Other nutritional; endocrine; and metabolic disorders	E832	Disorders of zinc metabolism
58	Other nutritional; endocrine; and metabolic disorders	E7521	Fabry (-Anderson) disease
58	Other nutritional; endocrine; and metabolic disorders	E8331	Familial hypophosphatemia
58	Other nutritional; endocrine; and metabolic disorders	E7421	Galactosemia
58	Other nutritional; endocrine; and metabolic disorders	E804	Gilbert syndrome
58	Other nutritional; endocrine; and metabolic disorders	E83111	Hemochromatosis due to repeated red blood cell transfusions
58	Other nutritional; endocrine; and metabolic disorders	E83110	Hereditary hemochromatosis
58	Other nutritional; endocrine; and metabolic disorders	E739	Lactose intolerance, unspecified
58	Other nutritional; endocrine; and metabolic disorders	E8581	Light chain (AL) amyloidosis
58	Other nutritional; endocrine; and metabolic disorders	E756	Lipid storage disorder, unspecified
58	Other nutritional; endocrine; and metabolic disorders	E881	Lipodystrophy, not elsewhere classified
58	Other nutritional; endocrine; and metabolic disorders	E882	Lipomatosis, not elsewhere classified
58	Other nutritional; endocrine; and metabolic disorders	E786	Lipoprotein deficiency
58	Other nutritional; endocrine; and metabolic disorders	E65	Localized adiposity
58	Other nutritional; endocrine; and metabolic disorders	E889	Metabolic disorder, unspecified
58	Other nutritional; endocrine; and metabolic disorders	E7212	Methylenetetrahydrofolate reductase deficiency
58	Other nutritional; endocrine; and metabolic disorders	E6601	Morbid (severe) obesity due to excess calories
58	Other nutritional; endocrine; and metabolic disorders	E662	Morbid (severe) obesity with alveolar hypoventilation
58	Other nutritional; endocrine; and metabolic disorders	E669	Obesity, unspecified
58	Other nutritional; endocrine; and metabolic disorders	E854	Organ-limited amyloidosis
58	Other nutritional; endocrine; and metabolic disorders	E8809	Other disorders of plasma-protein metabolism, NEC
58	Other nutritional; endocrine; and metabolic disorders	E8589	Other amyloidosis

58	Other nutritional; endocrine; and metabolic disorders	E7209	Other disorders of amino-acid transport
58	Other nutritional; endocrine; and metabolic disorders	E7439	Other disorders of intestinal carbohydrate absorption
58	Other nutritional; endocrine; and metabolic disorders	E701	Other hyperphenylalaninemias
58	Other nutritional; endocrine; and metabolic disorders	E668	Other obesity
58	Other nutritional; endocrine; and metabolic disorders	E8889	Other specified metabolic disorders
58	Other nutritional; endocrine; and metabolic disorders	R638	Other symptoms and signs concerning food and fluid intake
58	Other nutritional; endocrine; and metabolic disorders	E663	Overweight
58	Other nutritional; endocrine; and metabolic disorders	Z8639	Personal history of endo, nutritional and metabolic disease
58	Other nutritional; endocrine; and metabolic disorders	R636	Underweight
58	Other nutritional; endocrine; and metabolic disorders	E8350	Unspecified disorder of calcium metabolism

ICD diagnoses deleted based on clinical determination that they were not relevant to COVID-19 and/or insufficient counts in the population
