# Supplementary material for: Post-acute sequelae of SARS-CoV-2 with clinical condition definitions and comparison in a matched cohort
Source: Nat Commun. 2022 Oct 12;13:5822. doi: 10.1038/s41467-022-33573-6 (PMC9556630; doi:10.1038/s41467-022-33573-6)
Supplement: Supplementary file 3 — Reporting Summary [file 41467_2022_33573_MOESM3_ESM.pdf]

nature portfolio

Corresponding author(s): Michael Horberg
Last updated by author(s): 09/08/2022

# Reporting Summary

Nature Portfolio wishes to improve the reproducibility of the work that we publish. This form provides structure for consistency and transparency in reporting. For further information on Nature Portfolio policies, see our Editorial Policies and the Editorial Policy Checklist.

## Statistics

For all statistical analyses, confirm that the following items are present in the figure legend, table legend, main text, or Methods section.

| n/a | Confirmed | |
|---|---|---|
| ☐ | ☒ | The exact sample size (*n*) for each experimental group/condition, given as a discrete number and unit of measurement |
| ☐ | ☒ | A statement on whether measurements were taken from distinct samples or whether the same sample was measured repeatedly |
| ☐ | ☒ | The statistical test(s) used AND whether they are one- or two-sided<br>*Only common tests should be described solely by name; describe more complex techniques in the Methods section.* |
| ☐ | ☒ | A description of all covariates tested |
| ☐ | ☒ | A description of any assumptions or corrections, such as tests of normality and adjustment for multiple comparisons |
| ☐ | ☒ | A full description of the statistical parameters including central tendency (e.g. means) or other basic estimates (e.g. regression coefficient) AND variation (e.g. standard deviation) or associated estimates of uncertainty (e.g. confidence intervals) |
| ☐ | ☒ | For null hypothesis testing, the test statistic (e.g. *F*, *t*, *r*) with confidence intervals, effect sizes, degrees of freedom and *P* value noted<br>*Give P values as exact values whenever suitable.* |
| ☒ | ☐ | For Bayesian analysis, information on the choice of priors and Markov chain Monte Carlo settings |
| ☒ | ☐ | For hierarchical and complex designs, identification of the appropriate level for tests and full reporting of outcomes |
| ☐ | ☒ | Estimates of effect sizes (e.g. Cohen's *d*, Pearson's *r*), indicating how they were calculated |

*Our web collection on statistics for biologists contains articles on many of the points above.*

## Software and code

Policy information about availability of computer code

| | |
|---|---|
| Data collection | Data collection were performed using SAS software(version 9.4; Cary, North Carolina) and SQL developer version 17.3.1. |
| Data analysis | Analyses were performed using SAS software (version 9.4; Cary, North Carolina) with Tableau 2019.1 used to produce figure 2. Matching was performed using SAS data steps and Proc SQL. Unadjusted risk ratios with 95% confidence intervals (using the Wald Test method through SAS Proc Freq) were calculated for each CCS condition. |

For manuscripts utilizing custom algorithms or software that are central to the research but not yet described in published literature, software must be made available to editors and reviewers. We strongly encourage code deposition in a community repository (e.g. GitHub). See the Nature Portfolio guidelines for submitting code & software for further information.

## Data

Policy information about availability of data

All manuscripts must include a data availability statement. This statement should provide the following information, where applicable:

- Accession codes, unique identifiers, or web links for publicly available datasets
- A description of any restrictions on data availability
- For clinical datasets or third party data, please ensure that the statement adheres to our policy

As a clinical healthcare system, Kaiser Permanente (KP) is responsible for the confidentiality and protection of our members' data. As such, we are required by organizational policy to ensure outside access and/or procurement requests involving clinical data, regardless of data de-identification, are reviewed by the proper personnel within the Mid-Atlantic Permanente Research Institute (MAPRI) for appropriate research use, purpose, disclosure tracking, and potential for security risks.

Research data that is to be made publicly available for published manuscripts are required to remain behind Kaiser Permanente's firewall until it can be released to outside entities through secure file transfer protocols, provided all stipulations listed above for the data request are met.

All data were collected from internal Kaiser Permanente databases that are utilized for clinical care and claims. Information on the structure and name of the tables used in these databases are proprietary and cannot be shared publicly.

All requesters will be required to provide statement of need and comply with MAPRI policy and requirements. Failure to produce sufficient information and/or a request that doesn't meet MAPRI policy, may be denied.

## Human research participants

Policy information about studies involving human research participants and Sex and Gender in Research.

| | |
|---|---|
| Reporting on sex and gender | Sex was used as a matching criteria, but we did not exclude patients of a particular sex in this study. Sex is based on administrative sex which is as reported in our clinical electronic health records. Our patient population included 15,993 female COVID positive patients and 40,396 female COVID negative patients. We also included 12,125 male COVID positive patients and 29,897 male COVID negative patients. |
| Population characteristics | Our study population consisted of 28,118 PCR-positive patients and 70,293 PCR-negative patients. 1:3 case to control matching represented 66.8% of the identified cohort, followed by 16.2% with 1:2 and 16.8% with 1:1 matching. Overall, both case and control groups had ~57% female patients, a higher distribution of Black (~40%-43%) and Hispanic (~20%-24%) compared with white (~18-22%) patients, ~87% distribution less than 65 years old, and 30%-33% distribution of patients with a BMI ≥ 30 kg/m2 (refer to Table 5 in the manuscript). |
| Recruitment | Our study did not require recruitment |
| Ethics oversight | All patients in this study fall under a waiver of consent consistent with KPMAS guidance and protocol and approved by the Kaiser Permanente Mid-Atlantic States (KPMAS) IRB. |

Note that full information on the approval of the study protocol must also be provided in the manuscript.

# Field-specific reporting

Please select the one below that is the best fit for your research. If you are not sure, read the appropriate sections before making your selection.

☒ Life sciences ☐ Behavioural & social sciences ☐ Ecological, evolutionary & environmental sciences

For a reference copy of the document with all sections, see nature.com/documents/nr-reporting-summary-flat.pdf

# Life sciences study design

All studies must disclose on these points even when the disclosure is negative.

| | |
|---|---|
| Sample size | A base population of 31,390 total COVID-positive patients resulted. 28,118 COVID-positive and 70,293 COVID-negative patients resulted from our matching process. Given the sheer magnitude of COVID tested patients that met our criteria and were used in this study, it was determined the that our cohort size exceeded the necessary count for risk analysis. Our cohort is not a sample, but includes all possible Kaiser Permanente patients enrolled during our study period who had a COVID PCR test. |
| Data exclusions | We required patients to be >= 18 years and have a PCR result between 1/1/2020—12/ 31/2020. Our study was focused on the adult population and we wanted to ensure COVID infection was verified so we limited it to only those with a PCR result. We also limited our cohort to this particular period to avoid the influence of later variants and vaccinations. |
| Replication | It was determined that mutual exclusivity, based on time period of diagnosis, for specific conditions such as abdominal pain or fatigue may limit our incidence of those conditions in the acute and later time periods. We performed a sensitivity analysis whereby we re-ran our analysis but removed mutually exclusive requirements from symptom based conditions such as those. The results attenuated the increased risk ratios for PASC related conditions; however, none of the significant increased risk ratios changed statistical significance. Additionally, our work builds upon previous studies as mentioned throughout our manuscript, and our results are similar to those studies mentioned. Compared to other studies, our study does provide a larger and more diverse population; however, we cannot guarantee that the same results would be seen in other populations. |
| Randomization | Randomization was not required as we controlled for covariates using a matched cohort that utilized age, sex, testing month and medical center as match criterion |
| Blinding | This was a clinical study using actual clinical data known to Kaiser Permanente so blinding was not necessary |

# Reporting for specific materials, systems and methods

We require information from authors about some types of materials, experimental systems and methods used in many studies. Here, indicate whether each material, system or method listed is relevant to your study. If you are not sure if a list item applies to your research, read the appropriate section before selecting a response.

## Materials & experimental systems

| n/a | Involved in the study | |
|---|---|---|
| ☒ | ☐ | Antibodies |
| ☒ | ☐ | Eukaryotic cell lines |
| ☒ | ☐ | Palaeontology and archaeology |
| ☒ | ☐ | Animals and other organisms |
| ☒ | ☐ | Clinical data |
| ☒ | ☐ | Dual use research of concern |

## Methods

| n/a | Involved in the study | |
|---|---|---|
| ☒ | ☐ | ChIP-seq |
| ☒ | ☐ | Flow cytometry |
| ☒ | ☐ | MRI-based neuroimaging |
